# *De novo* and reference transcriptome assembly of transcripts expressed during flowering provide insight into seed setting in tetraploid red clover

**DOI:** 10.1038/srep44383

**Published:** 2017-03-13

**Authors:** Mallikarjuna Rao Kovi, Helga Amdahl, Muath Alsheikh, Odd Arne Rognli

**Affiliations:** 1 Department of Plant Sciences, Norwegian University of Life Sciences, NO-1432 Ås, Norway; 2 Graminor Breeding AS, Hommelstadvegen 60, NO-2322, Ridabu, Norway

## Abstract

Red clover (*Trifolium pratense* L.) is one of the most important legume forage species in temperate livestock agriculture. Tetraploid red clover cultivars are generally producing less seed than diploid cultivars. Improving the seed setting potential of tetraploid cultivars is necessary to utilize the high forage quality and environmentally sustainable nitrogen fixation ability of red clover. In the current study, our aim was to identify candidate genes involved in seed setting. Two genotypes, ‘Tripo’ with weak seed setting and ‘Lasang’ with strong seed setting were selected for transcriptome analysis. *De novo* and reference based analyses of transcriptome assemblies were conducted to study the global transcriptome changes from early to late developmental stages of flower development of the two contrasting red clover genotypes. Transcript profiles, gene ontology enrichment and KEGG pathway analysis indicate that genes related to flower development, pollen pistil interactions, photosynthesis and embryo development are differentially expressed between these two genotypes. A significant number of genes related to pollination were overrepresented in ‘Lasang’, which might be a reason for its good seed setting ability. The candidate genes detected in this study might be used to develop molecular tools for breeding tetraploid red clover varieties with improved seed yield potentials.

Red clover (*Trifolium pratense* L.) is a perennial forage legume species. It is outcrossing with a gametophytic self-incompatibility system, and it is cultivated mostly in temperate regions. Due to its nitrogen fixation ability, high protein content and digestibility, red clover is one of the most important forage legumes. Naturally, red clover is diploid (2n = 2X = 14); however, artificially induced tetraploid varieties (2n = 4X = 28) are also in commercial use. Tetraploid plants were first developed in 1939 by treating germinating seeds, young seedlings or apical meristem of diploids with the mitosis-inhibiting chemical colchicine^1^,^2^. New tetraploid plants can also be developed by treating diploid plants with nitrous oxide (N_2_O) and by gametic non-reduction^2^,^3^,^4^. However, red clover breeders develop new tetraploid varieties mainly by crossing plants from two or more tetraploid varieties or breeding lines.

The main advantages of cultivating tetraploid compared to diploid red clover are its higher forage yield, better persistency and tolerance to some diseases like *Sclerotinia trifoliorum* Eriks^1^,^4^,^5^,^6^. However, lower seed yield of tetraploid varieties is the major disadvantage compared to diploid cultivars^1^,^7^,^8^.

Seed yield of red clover, especially tetraploids, has not been improved in Scandinavia for a long time. The reasons for this are probably complex. A main reason is that several studies indicate that forage and seed yield are negatively correlated making seed yield improvement difficult^9^,^10^,^11^,^12^,^13^. Red clover is primarily grown for forage and forage yield is the main breeding goal; however, seed yield is crucial for the commercial value of new varieties^14^,^15^. The outcrossing nature and strong self-incompatibility system of red clover prevent the development of inbred lines and hybrids, thus only a proportion of the potential heterosis for seed yield can be captured in the usual synthetic varieties^13^,^15^.

Genomic resources related to seed yield are scarce in red clover compared to other model legume species. Currently, four genetic linkage maps for identification of markers linked to important traits have been developed in red clover^12^,^16^,^17^,^18^. Several QTL studies of seed yield and seed yield components have been conducted in species like white clover (*Trifolium repens* L.), soybean (*Glycine max* L.) and perennial ryegrass (*Lolium perenne* L.)^19^,^20^,^21^. However, so far, only one QTL study of seed yield has been performed in red clover, and this study identified 38 QTL^12^.

Rapid advancements in next generation sequencing (NGS) technology allow characterization and quantification of RNA through cDNA sequencing at massive scale^22^. A draft assembly of the red clover genome based on 16 different genotypes of red clover was recently published^23^. Furthermore, Yates *et al*.^24^ performed *de novo* transcriptome studies in red clover and provided insights into the drought response. De Vega *et al*.^25^ recently assembled a red clover genome to the chromosome level, estimating its size to be ~309 Mb. The group annotated 40,868 genes and identified clusters involved in forage quality and livestock nutrition.

With the availability of new genomic resources in red clover and the advancements in RNA-seq technologies, we performed both *de novo* and reference (red clover genome) based transcriptome analysis of the global transcriptome response during flower and seed development in two red clover genotypes with contrasting seed setting ability. The aim of this study was to identify molecular responses and to elucidate genes determining seed setting ability in red clover.

## Materials and Methods

### Plant material

In 2011, nine single plants of each of the low seed yielding variety ‘Tripo’, and the high seed yielding variety ‘Lasang’ were scored for the following seed yield components: number of flower heads per plant, number of florets per flower head, number of seed per flower head, fertility, seed weight per flower head, and length of the corolla tube^26^. The two lowest ranking plants of ‘Tripo’ and the two highest ranking plants of ‘Lasang’, for the majority of registered seed yield components, were selected for further analysis.

### RNA sampling

A total number of 12 flower buds, one bud from each of three flower development periods (early – 12^th^ of July, middle – 21^st^ of July and late – 27^th^ of July) from each of the four selected plants, were picked, flash frozen in liquid N_2_ and stored at −80 °C until RNA extraction. The frozen flower bud samples were crushed with a pestle and mortar. Using SIGMA SPECTRUM PLANT TOTAL RNA KIT (Sigma Life Science), total RNA was extracted from the 12 flower buds. On-Column DNAse Kit (Sigma Life Science) was used to remove DNA contamination. The quality and concentration of RNA were measured using the NANODROP (Nanodrop Technologies, Wilmington, DE, USA) and BIOANALYZER (Agilent Technologies, Palo Alto, CA, USA) equipment.

### RNA-seq library preparation and Illumina sequencing

Twelve flower bud RNA samples with RIN (RNA Integrity Number) values above 7 were used to construct separate cDNA libraries with fragment lengths of 200 bp (±25 bp). Single-end sequencing was performed at the Norwegian Sequencing Centre (NSC), University of Oslo, using the Illumina sequencing platform (HISEQ 2000) generating single-end reads with a length of 50 bp. The FastQC program (http://www.bioinformatics.babraham.ac.uk/projects/fastqc/) was used to analyse the quality of the raw sequencing reads.

### *De novo* transcriptome analysis

The *de novo* assembly was performed in a similar manner as described by Kovi *et al*.^27^. Briefly, adapter sequences and low quality reads were removed using the sickle program (https://github.com/najoshi/sickle/blob/master/README.md). The clean reads derived from the four individual genotypes named Tripo42 and Tripo55, Lasang77 and Lasang108, were used to construct separate *de novo* assemblies for each genotype using the Trinity assembler (release 2013-02-25)^28^. The *de novo* assembled transcriptome was then used as a reference to map the individual reads using the Bowtie program^29^. Transcript abundance was measured for each genotype and time point combination as the expected number of fragments per kilobase (kb) of transcript sequence per million mapped reads (FPKM)^30^ using RSEM version 1.1.11^31^.

### Identification of differentially expressed genes (DEGs), annotation and gene ontology (GO) analysis

The edgeR package^32^ in R program language (https://www.r-project.org/) was employed to identify DEGs and a false discovery rate (FDR) of 0.05 was further used to determine the significant DEGs. Transcripts showing differential expression at any flower development time-point were clustered using a K-means clustering algorithm. The annotation of the DEGs were performed using the Blast2GO program^33^. Initially, BLASTx was performed with an E-value threshold of 10e-06, followed by annotation with a cut-off value of 55 and GO weight Hsp-hit value of 20. The GO enrichment analysis was performed with a p-value of 0.01. The GO classification of DEGs in the two genotypes were generated using the WEGO program^34^. KEGG Pathway analysis was performed with the Blast2GO program^33^.

### Validation of *de novo* assembly by CEGMA

The CEGMA software (version 2.4)^35^ was used to evaluate the quality of the four transcriptome assembly datasets. Several genome and transcriptome assembly studies have used CEGMA for evaluating the quality of assemblies^27^. CEGMA detects the presence of 248 extremely conserved core eukaryotic genes (CEGs) and their coverage in transcriptome assemblies for evaluation of the completeness of the assembly.

### Red clover reference based transcriptome analysis, detecting DEGs and functional annotation

Using a reference-based approach, we mapped all the clean reads from the two genotypes (‘Tripo’ and ‘Lasang’) and the three time points (early, middle and late flower development) to the red clover reference genome^25^ using STAR, an ultrafast universal RNA-seq. aligner program^36^. The Cufflinks program^30^ was used to assemble the transcriptomes and to estimate the transcript abundance, followed by the cuffmerge and cuffdiff programs, which is included with the Cufflinks package. The Cuffmerge program merged the transcriptome assemblies from the three flower development time- points of each genotype for performing differential expression analysis. The Cuffdiff program compared the expression levels of genes and transcripts between the three time-points for each genotype, and detected genes that are up- or down-regulated between the time-points. The merged GTF files obtained from the Cuffmerge program was used in the TransDecoder program^37^ to identify the coding regions within transcripts. The longest homology coding sequences obtained from TransDecoder were blasted against the Viridiplantae database extracted from NCBI to find the gene names for the coding sequences. Further annotation was performed using the SWISS-PROT database. GFF3 (generic feature format) annotation file describing genomic features, was generated using in-house developed python scripts.

### Comparison of significant DEGs to seed yield related QTL

To compare the DEGs with the QTL for seed yield and seed yield traits described by Hermann *et al*.^12^, we identified flanking SSR markers associated with the QTL and downloaded the marker sequences from the NCBI database. The chromosome locations of markers and DEGs were identified using the BLAST program with the marker sequences and DEG sequences as the query and the red clover genome sequence^25^ as the subject. A physical map was created based on the physical location of the DEGs in the red clover genome by the MapDraw software^38^. Briefly, all the physical location (bp) of DEGs were converted to centimorgan (cM) by an average of 450 kb/cM in red clover and spanned 440 cM across seven linkage groups (LGs), approximately similar to 444 cM of Hermann *et al*.^12^.

## Results

### *De novo* assembly

**Figure 1:**
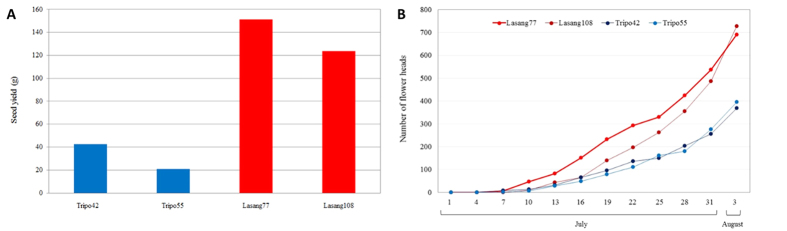
(**A**) Cumulative curve for number of flower heads for four tetraploid red clover genotypes: two low seed yielding ‘Tripo’ genotypes and two high seed yielding ‘Lasang’ genotypes. The x-axis indicates the date when counting of flower heads was performed. (**B**) Seed yield per plant in low seed yielding tetraploid red clover cv. ‘Tripo’ and high seed yielding tetraploid red clover cv. ‘Lasang’.

The low seed yielding ‘Tripo’ and the high seed yielding ‘Lasang’ genotypes (Fig. 1) were sequenced and characterized by the *de novo* transcriptome assembly (Table 1). A total number of 218 million reads of 50 bp were generated for the four genotypes (Tripo42, Tripo55, Lasang77 and Lasang108). 112 million reads were from the ‘Tripo’ genotypes and 106 million reads from the ‘Lasang’ genotypes. Individual transcriptome assemblies were generated for each genotype. The numbers of contigs observed in Lasang108 and Lasang77 were 80,328 (N50 of 930 bp) and 83,489 (N50 of 982 bp), respectively, while in Tripo42 and Tripo55, they were 84,545 (N50 of 1016 bp), and 84,442 (N50 of 982 bp), respectively. The longest contig sizes were 7469, 7295, 7447 and 7339 bp for Lasang108, Lasang77, Tripo42 and Tripo55, respectively. CEGMA analysis determined the complete CEGs (Core Eukaryotic Genes) in Lasang108, Lasang77, Tripo42 and Tripo55 transcriptome assemblies to be 89.11, 92.34, 92.34 and 92.34%, respectively, while the percentage of partially complete CEGs ranged from 97.18 to 97.98 (Table 2). The average number of orthologues per CEG in the four assemblies ranged from 3.18 to 3.30, while the percentage of CEGs that had more than one orthologue ranged from 89.59 to 95.20 (Table 2).

### DEGs identified by *de novo* and reference based methods

**Figure 2:**
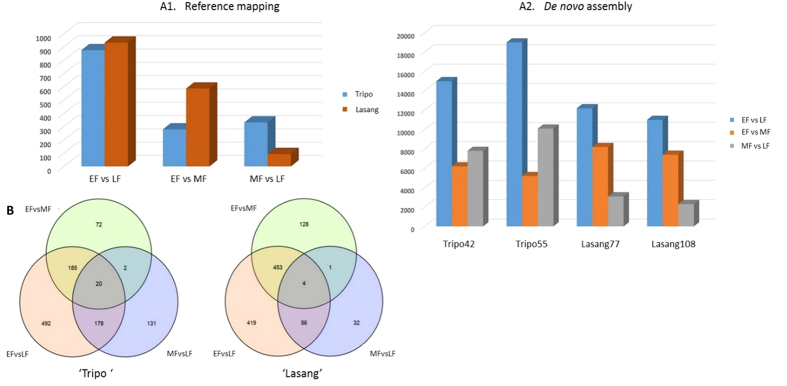
The number of differentially expressed transcripts identified using reference based assembly (**A1**) and *de novo* based assembly (**A2**). (**B**) Venn diagrams represents the number of up- and down-regulated transcripts that were common and specific for the pairwise comparisons using the reference based assembly in pairwise comparisons between EF vs LF (early vs. late flowering), EF vs MF (early vs. mid flowering), and MF vs LF (mid vs. late flowering) in genotypes ‘Tripo’ and ‘Lasang’ using a false discovery rate (FDR) of <0.05.

Clean reads from each sample were mapped onto their respective genotype specific *de novo* assemblies and to the reference genome (red clover genome sequence) to estimate the expression levels of transcripts at different flower development time-points, early (EF), middle (MF) and late (LF) flower development. The DEGs identified in a series of pairwise comparisons between the three flower development time-points EF-LF, EF-MF and LF-MF were 15,000, 7,204 and 7,903, respectively, in Tripo42; 18,105, 6,050 and 10,100, respectively, in Tripo55; 12,040, 8,426 and 2,304, respectively, in Lasang77; and 10,986, 7,492 and 2,430, respectively, in Lasang108 with a false discovery rate (FDR) <0.05 (Fig. 2B). In the reference-based analysis, 875 and 932 DEGs were observed between EF-LF samples; 279 and 586 between EF-MF samples and 331 and 93 between MF-LF samples in the ‘Tripo’ and ‘Lasang’ genotypes, respectively, including up- and down-regulated transcripts (Fig. 2A).

**Figure 3:**
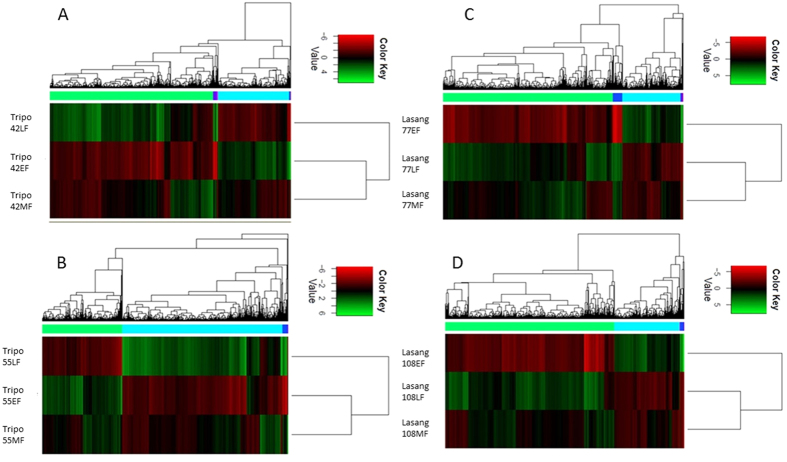
Heat maps of differentially expressed genes detected using *de novo* assemblies for each genotype and grouped according to their expression patterns. Y-axis represents the experimental conditions. (**A**) Tripo42, (**B**) Tripo55, (**C**) Lasang77, and (**D**) Lasang108. EF; early flowering, MF; middle flowering. LF; late flowering.

To determine the sample relations, differential expression data from the edgeR program were used to generate heat maps (Fig. 3). EF and MF grouped together in the low seed yielding ‘Tripo’ genotypes, while MF and LF grouped together in the high seed yielding ‘Lasang’ genotypes, indicating that unique genes expressed during late flower development (LF) in ‘Tripo’ and early flower development (EF) in ‘Lasang’ were playing major roles in their flowering and seed setting abilities.

### Blast, annotation and GO of differentially expressed genes

**Figure 4:**
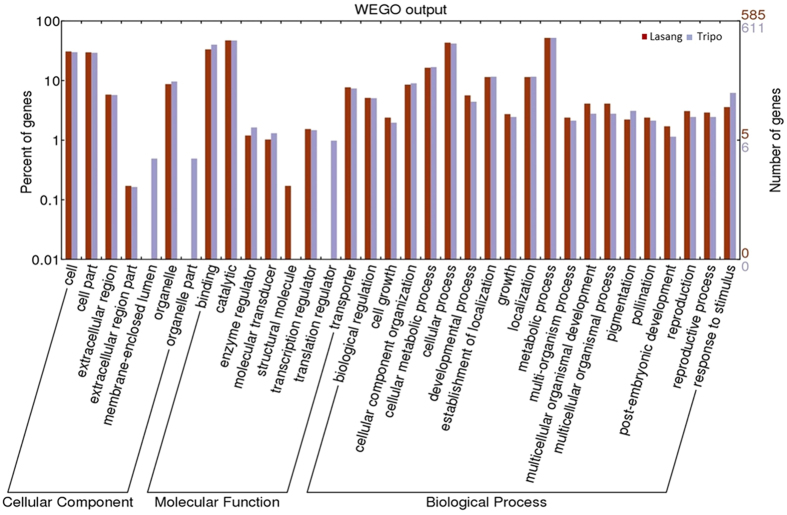
Gene ontology classifications of differentially expressed genes observed during pairwise comparisons of ‘Tripo’ and ‘Lasang’ genotypes generated by the WEGO tool (http://wego.genomics.org.cn/cgi-bin/wego/index.pl) using the newest GO archive provided. The results are distributed in three main GO categories: cellular component, molecular function and biological process. The right y-axis indicates the number of genes in a GO category for each genotype. The left y-axis indicates the percentage of a specific category of genes in the respective main categories for each genotype.

BLASTx was performed for all the DEGs against the Viridiplantae database derived from NCBI. Approximately 80% of the DEGs had blast hits and 60% were annotated using the Blast2GO program^33^. The top blast hit species were *Trifolium subterraneum*, followed by *Medicago truncatula*. Bboth species are closely related to red clover. Gene ontology (GO) classification of DEGs of ‘Tripo’ and ‘Lasang’ were represented as three main GO categories, i.e. cellular component, molecular function and biological process in a histogram (Fig. 4) using the WEGO (Web Gene Ontology Annotation Plot) graphical tool^34^. GO comparisons between ‘Tripo’ and ‘Lasang’ showed some differences regarding the cellular component and molecular function categories, while relatively small differences were observed for the biological process category. DEGs involved in membrane-enclosed lumen and translation regulator were present only in ‘Tripo’, while DEGs involved in structural molecule were present only in ‘Lasang’.

**Figure 5:**
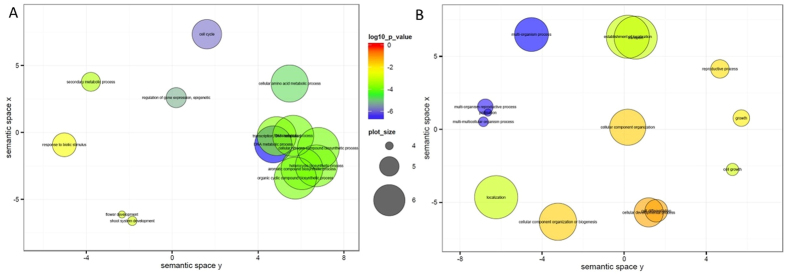
Gene ontology (GO) enrichment analysis by Fischer’s exact test. The scatterplot of GO terms which are associated with differentially expressed genes in ‘Tripo’ (**A**) and in ‘Lasang’ (**B**) shows the cluster representatives (i.e. terms remaining after the redundancy reduction) in a two dimensional space derived by applying multidimensional scaling to a matrix of the GO terms’ semantic similarities. Bubble color indicates p-value (-log10 p-value); size indicates the frequency of the GO term in the underlying GOA database (bubbles of more general terms are larger). In ‘Tripo’ (low seed yield) (**A**) has higher representation of GO of flower and shoot system development. In ‘Lasang’ (high seed yield) (**B**) has higher representation of GO for pollination or pollen-pistel interactions (multi-organism process).

Over- or underrepresented GO terms were determined using Fischer’s exact test in the Blast2GO program, and the REVIGO tool for reducing and visualising gene ontologies^39^. Six GO terms were enriched when compared ‘Tripo’ and ‘Lasang’ genotypes. Out of these, four GO terms, i.e. plasma membrane, pollination, transport and Golgi apparatus were overrepresented in the high seed yielding ‘Lasang’ genotypes. Transcripts assigned to DNA metabolic processes and nucleic acid binding were overrepresented in the low seed yielding ‘Tripo’ genotypes (Fig. 5, Supplementary Figure 1).

Several genes, putatively involved in flower and seed development were detected in these studies, e.g. walls are thin related protein (WAT1), tubby-like F-box protein, gibberellin (GA) 2–beta-dioxygenase, putative aquaporin NIP4-1, zinc finger protein 4, which all were significantly upregulated from the EF to the MF stage, and significantly downregulated from MF to LF (Table 3, Fig. 2). Ethylene-responsive transcription factor (ERF106), probable inorganic phosphate transporter 1–4 (OsPht1;4) were significantly downregulated from the EF to the MF stage, while they were upregulated from MF to LF stage (Table 3, Fig. 2). Furthermore, the Kyoto encyclopedia of genes and genomes (KEGG) database detected different pathways between ‘Tripo’ and ‘Lasang’ at EF-MF and MF-LF stages. In total, 1196 DEGs were involved in 87 pathways (Supplementary Table 1). Pathways with highest representation among the genes were involved in starch and sucrose metabolism (4.84%, 58 genes), pentose and glucoronate interconversions (2.84%, 34 genes), phenylpropanoid biosynthesis (2.75%, 33 genes), purine metabolism (2.34%, 28 genes) and thiamine metabolism (2%, 24 genes).

### DEGs compared to the seed yield QTL

**Figure 6:**
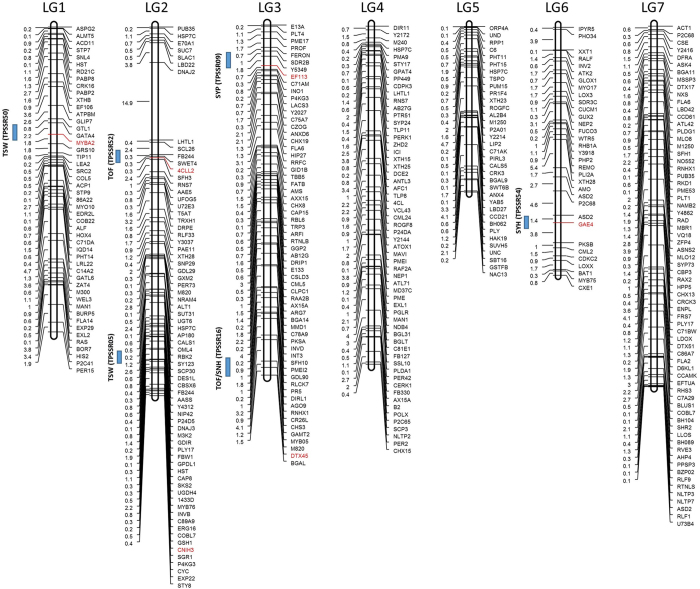
Comparative mapping of significant differentially expressed genes (DEGs) detected in this study to the red clover seed yield related QTL (Hermann *et al*., 2006). The distribution of DEGs on the seven linkage groups (LG) of red clover. The SSR markers (denoted in brackets) (Hermann *et al*., 2006), linked to QTL (denoted in bars) which are co-located to the DEGs are highlighted. In LG1, MYBA2 mapped to the QTL for thousand seed weight (TSW). In LG2, 4CLL2 and CNIH3 mapped to QTL for time of flowering (TOF) and TSW. In LG3, EF113 mapped to QTL for seed yield per plant (SYP). In LG6, GAE4, mapped to the QTL for seed yield per head (SYH).

The DEGs identified in this study were compared to seed yield related QTL in order to see if any of the genes identified are co-located with the seed yield QTL as described by Hermann *et al*.^12^. Out of 15 SSR markers flanking the seed yield QTL, six SSR markers are located in the corresponding regions as six DEGs detected in this study positioned on four linkage groups (Fig. 6). The six DEGs are myb-related protein MYBAS2, 4-coumarate–CoA ligase-like 2, protein cornichon homolog 3, ethylene-responsive transcription factor ERF113, protein DETOXIFICATION 45, and UDP-glucuronate 4-epimerase 4.

## Discussion

### Comparative analysis between *de novo* and reference based transcriptome assays

When a reference genome is available, reference-based approaches have been considered more effective than *de novo* assembly (Martin and Wang, 2011), but very few studies have compared the two strategies^27^,^40^. Moreover, it is important to see whether the *de novo* assembly can detect the same genes and the molecular responses even in the absence of a reference genome. In the present transcriptome analysis of red clover, we compared both strategies. The CEGMA analysis showed that the *de novo* assemblies were very complete in terms of gene content since they captured high percentages of ultra-conserved CEGs in all assemblies of the ‘Tripo’ and ‘Lasang’ genotypes. *De novo* and reference-based (red clover reference genome) gene expression data indicated that genes expressed during the early flower development stage (EF) in ‘Lasang’ and during the late flower development stage (LF) in ‘Tripo’ might play key roles in their differential seed setting abilities. In both *de novo* and the reference-based mapping, the pattern of the differentially expressed transcripts was similar. A larger number of differentially expressed transcripts was observed in early vs late flower development stage than in middle vs late and middle vs early stage (Figs 2 and 3). This might be due to the presence of several differentially expressed transcripts at all three stages. In addition, there was a larger number of differentially expressed transcripts at the early vs middle flower development stage in ‘Lasang’ than in ‘Tripo’, whereas there was more differentially expressed transcripts at the early vs late stage in ‘Tripo’ compared to ‘Lasang’ (Fig. 2). Furthermore, we found the proportion of differentially expressed transcripts to be higher in *de novo* compared to the reference based mapping, which is similar to the findings of Kovi *et al*.^27^. The trinity *de novo* assembler yield more transcripts due to the lack of strand-specific information. However, most of the differentially expressed genes identified in both these methods were related to the *Medicago truncatula* (Supplementary Figure 2), which is the most closely related species to red clover. Furthermore, both methods identified many similar candidate genes putatively involved in flower and seed development (Table 3), thus demonstrating the potential of the *de novo* method of capturing genes even in the absence of a reference genome. This comparative analysis study might be very useful for the researchers working on orphan species with no reference genome.

### Potential candidate genes involved in flower and seed development

Several genes putatively involved in flower development were detected in this study (Table 3). WAT1 related protein is a cell wall protein mainly responsible for transmembrane transporter activity (http://www.uniprot.org/uniprot/Q94AP3). Ranocha *et al*.^41^ reported that stem apices in the mutant *wat1* produced significantly lower seed yields in *Arabidopsis thaliana* compared to wild type stem apices. It might be that the downregulated expression of this gene in ‘Tripo’ flower buds in the early and middle flower development periods, negatively affected its seed setting ability and thus seed yield.

Tubby-like proteins are involved in abscisic acid (ABA) signaling pathways and plays a key role in seed germination and early seed growth^42^. In a recent study, Verma *et al*.^43^ identified a tubby-like F-box protein as a potential candidate gene for the seed weight QTL *qSW* in chickpea (*Cicer arietinum* L.). Gibberellin 2–beta-dioxygenase was highly expressed in EF and MF. According to Xue *et al*.^44^, genes that encodes gibberellin 2-beta-dioxygenase 1 were highly expressed in rice embryo.

NIP4-1 belongs to the aquaporin gene family, which are small integral membrane proteins that facilitate water and solute movement across different tissues throughout development and growth^45^. Regulation of water and nutrient state is very relevant for pollen development, pollen tube growth and germination^46^. Recently Di Giorgio *et al*.^47^ showed that NIP4-1 and NIP4-2 are required for pollen development and pollination in *Arabidopsis thaliana*. Furthermore, single *nip4*;*1* mutant plants showed a significantly higher frequency of abnormal, stunted siliques and fewer seeds when compared with the wild type^47^. This indicate that NIP4-1 plays a prominent role in determining seed yield. In our studies, the significant upregulation of this gene in ‘Lasang’ during the EF and MF stages might play a key role in determining the better seed yielding capacity of this cultivar.

Zinc finger proteins (ZFP) play an important role in various biological functions, such as plant growth and development (flower, shoot, seed, pistil and leaf)^48^,^49^. Recently it was found that ZFP3, ZFP4 and the related ZFP subfamily of zinc finger factors regulate light and ABA responses during germination and early seedling development^50^. Higher expression of ZFP4 during the EF and MF stages indicate that it might be important for seed setting in our tetraploid red clover genotypes.

The gene ERF106 belongs to the APETALA2 (AP2) gene family, which controls seed weight (Ohto *et al*.)^51^, was overexpressed during the EF and MF stages in ‘Lasang’ flower buds. APETALA2 influences the development of embryo, endosperm and seed coat^51^. According to Xue *et al*.^44^, genes involved in ethylene mediated signaling were highly expressed in rice developing seeds.

The rice gene OsPht1,4 belongs to a group of genes that regulate phosphorus homeostasis in plant cells^52^. Jia *et al*.^53^ reported that suppression of OsPT4 in rice resulted in lower P content in unfilled rice grains, which again resulted in lower seed yields. This gene was overexpressed during the MF and LF stages in ‘Lasang’ flower buds indicating its positive effect on seed yield. This gene is also involved in the embryo development in rice^54^.

### GO differences in ‘Tripo’ and ‘Lasang’

Gene ontology (GO) has provided a way of consistently describing genes and proteins to computationally process data at the functional level^34^,^39^. Gene ontology (GO) comparisons between the two genotypes showed differences regarding the cellular component and molecular function categories (Fig. 4). Six GO terms were enriched between these two genotypes. Out of these four GO terms, plasma membrane, pollination, transport and Golgi apparatus were overrepresented in the high seed yielding ‘Lasang’. Transcripts assigned to DNA metabolic processes and nucleic acid binding were overrepresented in the low seed yielding ‘Tripo’ (Fig. 5; Supplementary Figure 1). Genes, such as pollen-specific leucine-rich repeat extensin-like protein 1-like (PEX1), pollen profiling variant 1, phd finger protein male sterility 1–like (MS1-PHD), and polypyrimidine tract-binding protein, were observed in the pollination GO term. PEX1 reported to be involved in reproduction with in the pollen tube wall during its rapid growth^55^. Another gene, MS1-PHD encodes a PHD-type transcription factor and regulates pollen and tapetum development and pollen wall biosynthesis^56^. In the GO term plasma membrane, genes like aberrant pollen transmission (APT1), flotillin-like protein, sodium transporter hkt1-like were observed. Xu and Dooner^57^ showed that the APT1 protein is involved in membrane trafficking and is required for the high secretory demands of tip growth in pollen tubes. Most of the overrepresented genes are linked to pollen development, which is crucial for fertility and seed setting, thus likely involved in determining the higher seed yield capacity of ‘Lasang’ compared with ‘Tripo’.

### Validation of the DEGs by comparing to previous red clover seed yield QTL

Comparative mapping studies are powerful tools to validate the detected DEGs by comparing the sequences of the genes to the sequences of markers located inside or flanking QTL^58^. The physical map of DEGs was created based on their physical locations (bp) in the red clover genome. However, there is no constant ratio to convert between bp and cM, as some regions of the genome with frequent recombination have fewer bp per cM than regions with low recombination. The best approach might be to pick the most detailed genetic map (in our case ref. 12), fetch the sequences for each SSR marker on the map, and BLAST these marker sequences against the genome sequence (red clover genome). From the BLAST analysis, we were able to translate each of these cM distances into a bp distance between the points of alignment from the markers to the chromosome, thus calculated as 450 kb/cM. A similar approach was carried out in *Arabidopsis* by estimating genetic distance as 250 kb/cM^59^. In this study, we detected six DEGs that mapped to the seed yield QTL regions identified by Hermann *et al*.^12^, and positioned on linkage groups LG1, LG2, LG3 and LG6 (Fig. 6). Among them, MYB transcription factors play a key role in plant development, pollen development^60^, pollen tube differentiation^61^, floral initiation and seed development^62^. The gene ‘protein cornichon homolog’ belongs to a conserved protein family found in eukaryotes demonstrated to participate in the selection of integral membrane proteins as cargo for their correct targeting^63^. Further, Man *et al*.^58^ detected protein cornichon homolog as a potential gene encoding for the yield related QTL in cotton. 4-coumarate–CoA ligase-like 2, belongs to a group of essential enzymes involved in the phenylpropanoid-derived compound (PDC) pathway, which generates various secondary compounds like lignin, anthocyanins, and isoflavonoids. Doughty *et al*.^64^ suggests that flavonoids may play a fundamental role in regulating communication between the seed coat and the endosperm also.

## Conclusions

In this study, transcriptome analysis was conducted for cv. ‘Tripo’ with inferior seed setting ability and two from cv.‘Lasang’ with improved seed setting ability, and several DEGs were identified. Many genes related to pollination, flower and seed development were upregulated during the early to middle (EF-MF) flower development stage in the ‘Lasang’ and downregulated during the middle to late (MF-LF) flower development stage in the ‘Tripo’, indicating their major role in determining seed setting and potential seed yield. GO enrichment analysis further confirmed that plasma membrane, pollination, transport and Golgi apparatus related genes are overrepresented in the ‘Lasang’. Further, comparative mapping, co-located six seed yield related QTL to the six DEGs on the same linkage groups, thus validating the detected DEGs in this study. Putative candidate genes detected in this study might provide a basis for future functional genomics research in understanding the biology of seed yield in red clover. Loss-of-function techniques like RNA interference methods can further be used to understand the role of these genes in the seed setting.

## Additional Information

**Accession codes:** The raw Illumina sequencing data generated in this study were deposited in the EMBL-EBI ArrayExpress Archive, under accession number E-MTAB-5117. *De novo* transcriptome assemblies of four red clover genotypes generated by trinity program are deposited in DRYAD Digital Repository along with the GFF3 annotation files and script. (http://datadryad.org/resource/doi:10.5061/dryad.0bk52).

**How to cite this article:** Kovi, M. R. *et al*. *De novo* and reference transcriptome assembly of transcripts expressed during flowering provide insight into seed setting in tetraploid red clover. *Sci. Rep.* **7**, 44383; doi: 10.1038/srep44383 (2017).

**Publisher's note:** Springer Nature remains neutral with regard to jurisdictional claims in published maps and institutional affiliations.

## Supplementary Material

Supplementary Information

## Figures and Tables

**Table 1:** Characteristics of the *de novo* transcriptome assemblies.

Genotypes	Total number of contigs	N50 (bp)	Maximum contig length (bp)	Total number of reads
Lasang108	80,328	930	7469	51,000,000
Lasang77	83,489	982	7295	55,000,000
Tripo42	84,545	1016	7447	57,000,000
Tripo55	84,442	982	7339	55,000,000

**Table 2:** Results of CEGMA analysis for *de novo* assembly validation.

Out of 248 CEGs^1^	Lasang108	Lasang77	Tripo42	Tripo55
% of fully represented	89.11	92.34	92.34	92.34
% of at least partially represented	97.98	97.58	97.18	97.98
Average number of orthologues per CEG	3.19	3.28	3.18	3.30
% of detected CEGs with more than 1 orthologue	89.59	95.20	90.39	89.96

^1^CEGs: Core Eukaryotic Genes.

**Table 3:** List of differentially expressed genes that can be considered as potential candidate genes involved in seed setting in two red clover genotypes, ‘Tripo’ and ‘Lasang’.

Sequence ID	Description	Chromosome position	LogFC EF/MF	LogFC MF/LF
XLOC_000673	Probable inorganic phosphate transporter 1–4	LG1:16251287–16253813	−100.00	100.00
XLOC_001313	Polyadenylate-binding protein 8	LG1:2381924–2385791	−4.37	−2.69
XLOC_001371	Polyadenylate-binding protein 2	LG1:3705560–3712487	4.44	−1.06
XLOC_002319	Peroxidase 15	LG1:27442419–27444838	−4.42	−4.35
XLOC_002704	Ethylene-responsive transcription factor ERF106	LG1:5667372–5667944	−100.00	100.00
XLOC_005163	Uncharacterized protein	LG2:31978172–31979622	−4.12	−0.79
XLOC_008531	Long chain acyl-CoA synthetase 3	LG3:4387754–4394714	4.78	−0.45
XLOC_009167	Type III polyketide synthase A	LG3:18921285–18925387	−5.42	−3.29
XLOC_009463	Gibberellic acid methyltransferase 2	LG3:26837375–26842634	−4.62	−2.65
XLOC_009550	Protein DETOXIFICATION 45, chloroplastic	LG3:29591163–29602019	5.69	−2.03
XLOC_013980	Tubby-like F-box protein 13	LG4:5131256–5132175	100.00	−2.95
XLOC_015206	Tubby-like F-box protein 11	LG4:5130358–5131150	100.00	−2.86
XLOC_016540	Non-specific lipid-transfer protein C6	LG5:486967–488591	−6.78	−5.40
XLOC_016934	Potassium transporter 19	LG5:12411777–12412424	100.00	−2.14
XLOC_017036	Inorganic phosphate transporter 1–1	LG5:725199–729206	4.13	−0.70
XLOC_019592	Cucumisin	LG6:5874355–5878558	6.48	−2.67
XLOC_020680	Alpha-L-fucosidase 3	LG6:8011079–8011470	100.00	−0.23
XLOC_022367	Elongation factor TuA, chloroplastic	LG7:23203090–23207956	5.30	−2.26
XLOC_022717	Uncharacterized protein	LG7:1020381–1022267	100.00	−1.77
XLOC_024498	Zinc finger protein 4	LG7:11459754–11460460	100.00	−0.20
XLOC_025967	Putative aquaporin NIP4-1	scaf_10543:0–1249	100.00	−2.40
XLOC_028325	Probable E3 ubiquitin-protein ligase ARI10	scaf_1364:21411–23094	100.00	−5.16
XLOC_028939	Glycerophosphodiester phosphodiesterase GDPDL4	scaf_14591:517–949	100.00	−1.10
XLOC_029741	Cystinosin homolog	scaf_15837:737–851	100.00	−2.98
XLOC_029976	Nudix hydrolase 1	scaf_1621:8252–8547	100.00	−1.89
XLOC_034559	Probable magnesium transporter NIPA2	scaf_25880:5–754	100.00	−1.62
XLOC_037847	1-aminocyclopropane-1-carboxylate oxidase homolog 1	scaf_34610:27–594	100.00	−0.94
XLOC_039083	Probable 2-oxoglutarate/Fe(II)-dependent dioxygenase	scaf_3804:1914–2262	100.00	−3.64
XLOC_039720	17.2 kDa class II heat shock protein	scaf_4026:0–243	100.00	−5.90
XLOC_040357	B3 domain-containing protein	scaf_440:86915–89742	100.00	−3.55
XLOC_041752	Cytochrome P450 94B1	scaf_521:81827–84098	5.26	−1.65
XLOC_043901	L-type lectin-domain containing receptor kinase VIII.2	scaf_6561:0–1615	−100.00	100.00
XLOC_044539	Gibberellin 2-beta-dioxygenase	scaf_702:17734–18233	100.00	−0.65
XLOC_045146	WAT1-related protein	scaf_743:105103–107134	100.00	−2.56
