# Supplementary material for: De novo and reference transcriptome assembly of transcripts expressed during flowering provide insight into seed setting in tetraploid red clover
Source: Sci Rep. 2017 Mar 13;7:44383. doi: 10.1038/srep44383 (PMC5347025; doi:10.1038/srep44383)
Supplement: Supplementary Information [file srep44383-s1.pdf]

De novo and reference transcriptome assembly of transcripts expressed during flowering provide insight into seed setting in tetraploid red clover

Mallikarjuna Rao Kovi[1], Helga Amdahl[1,2], Muath Alsheikh[1,2,§] and Odd Arne Rognli[1]

[1]*Department of Plant Sciences, Norwegian University of Life Sciences, NO-1432 Ås, Norway*

[2]*Graminor Breeding AS, Hommelstadvegen 60, NO-2322, Ridabu, Norway*

[§]Corresponding author: muath.alsheikh@graminor.no

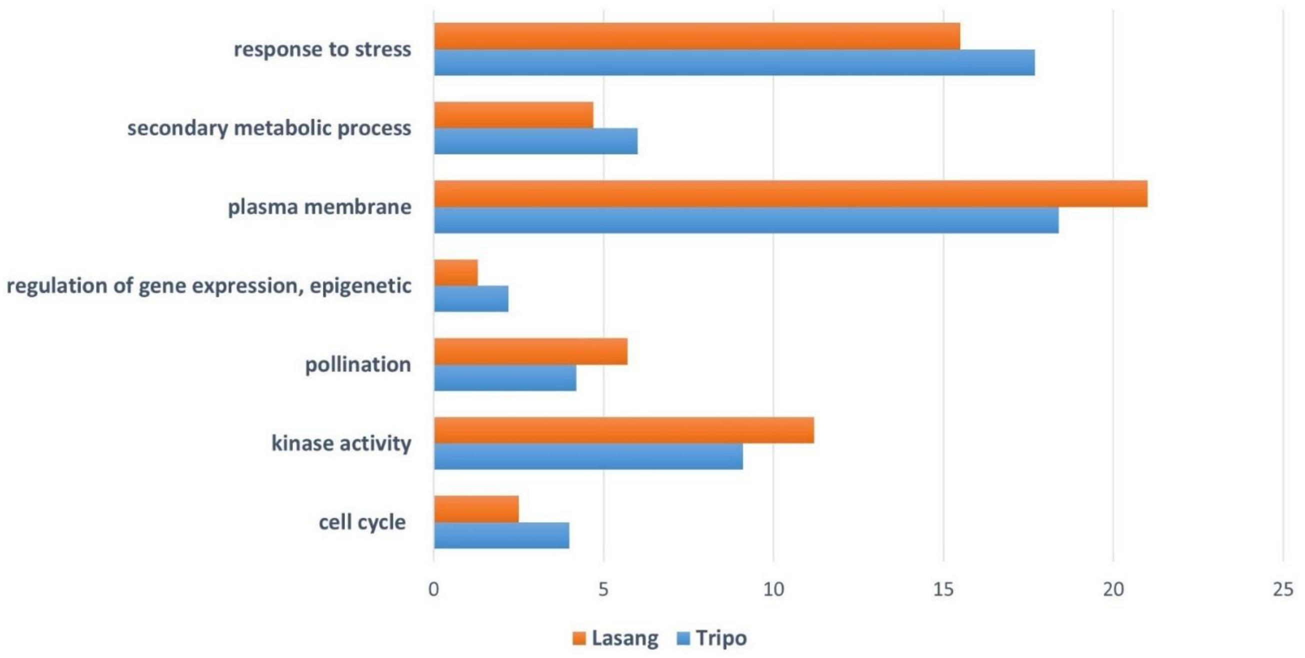

Supplementary Figure S1. Annotation differences between 'Lasang' and 'Tripo' genotypes detected by Fischer's exact test.

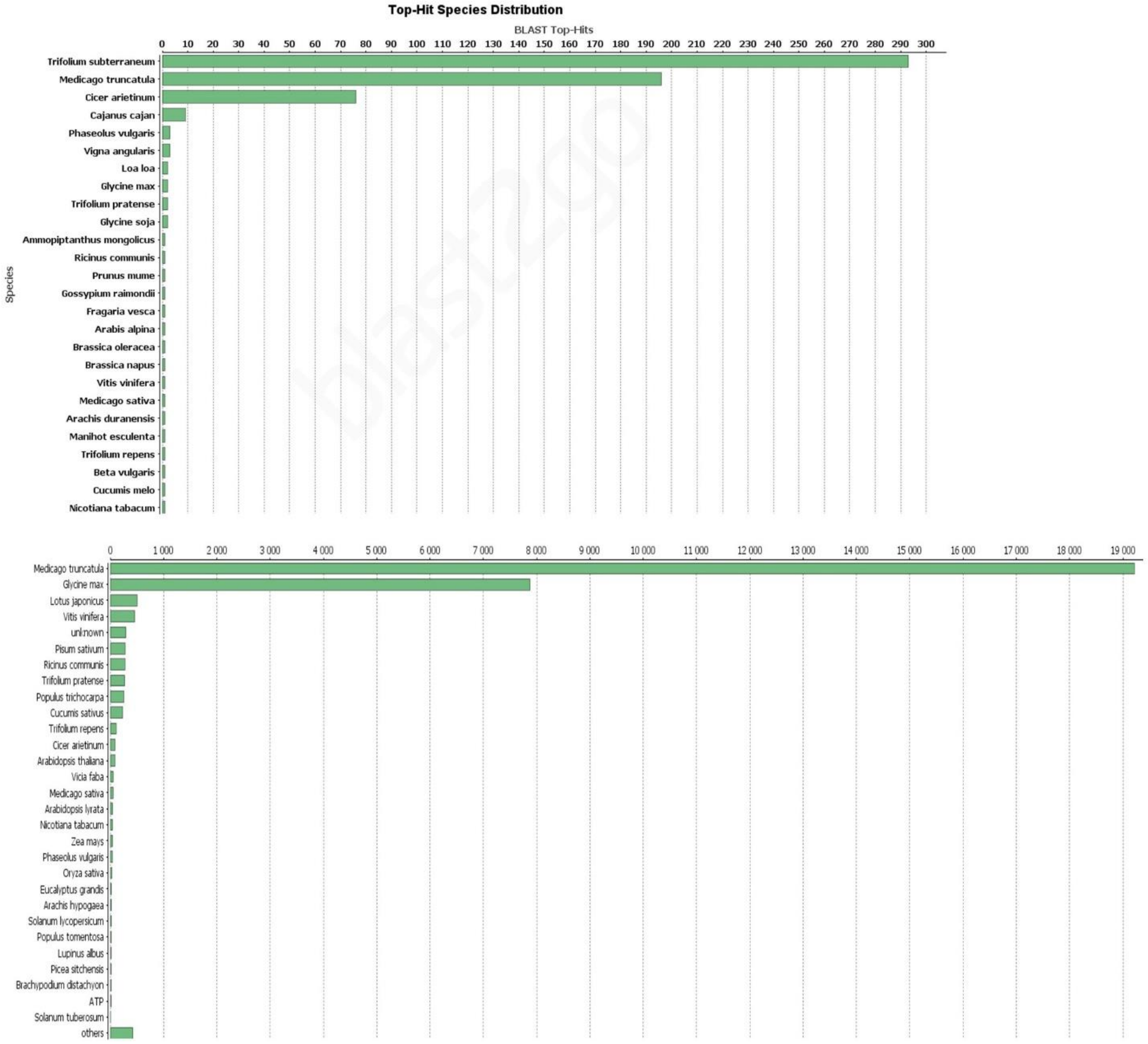

Supplementary Figure S2. The top blast hit-species distribution in reference based and *de novo* based assembly.

Supplementary Table S1. List of pathway-enriched differentially expressed genes in top ten pathways between ‘Tripo’ and ‘Lasang’ at EF-MF and MF-LF stages.

Pathway	Differentially expressed genes (No./%)	Pathway ID
Starch and sucrose metabolism	58 (4.84%)	map00500
Pentose and glucuronate interconversions	34(2.84%)	map00040
Phenylpropanoid biosynthesis	33 (2.75%)	map00940
Purine metabolism	28 (2.34%)	map00230
Thiamine metabolsim	24 (2.00%)	map00730
Amino sugar and nucleotide sugar metabolism	23 (1.92%)	map00520
Drug metabolism - other enzymes	22 (1.83%)	map00983
Aminobenzoate degradation	18 (1.50%)	map00627
Biosynthesis of antibiotics	18 (1.50%)	map01130
Flavonoid biosynthesis	18 (1.50%)	map00941
